# With a British Field Ambulance in August

**Published:** 1914-10-17

**Authors:** 


### WITH A BRITISH FIELD AMBULANCE IN AUGUST.

### By an Army Medical Officer at the Front.

There seems more likelihood of getting a letter through safely, and there certainly is more time to write one, than has been the case. I have sent off several postcards and letters. I have also asked officers that I have befriended to write home to announce that I am still alive and well. I have been reported dead so frequently that I have feared the rumour might reach England. I do not mean that I have been officially returned as dead, but in my various adventures I have been so far lost, at least twice, that it began to be put about that I was "done for." But, on the contrary, I am exceedingly fit and well—never better. I have enjoyed my adventures, and have seen a great deal of the game. Incidentally I have marched and travelled over a large tract of the North of France and the frontier of Belgium.

### French and English Organisation.

I will not dwell on the labour of loading wagons into French rolling-stock on sidings quite unsuited for the purpose. It was very heavy. Work that in England was performed easily in twenty-five minutes here only is to be accomplished in four or five hours with intense exertions. It has been a surprise to me to discover that the English Army is years ahead of the French Army in organisation and efficiency. Had we the same numbers or half the numbers, we could walk across Europe without difficulty. It is astonishing to find that a military nation should not have provided facilities for running guns and wagons on to trains. We got to a rail-head, after a long journey, not far from the Belgian Frontier. I am vague about names of set purpose, because it is considered desirable that strictest secrecy be maintained still on all that concerns our movements. We had great difficulty in unloading our wagons again, but I invented a method of fixing ramps on to the ends of groups of wagons and running the wagons down them. We held back by means of drag-ropes I borrowed off some A.V.C., and so got all down without accident to wagon or man. Immediately on detraining we set off to march about eleven miles with a column of troops.

### A Night Billet on the Frontier.

We got to billets in the dark. Our men were much tired, because we had been marching from 9.30 p.m. the day before. We had had four hours' arduous labour, from 11 p.m. to 3 a.m., loading into trucks, many hours crowded in a train, and three hours in great heat unloading. The enthusiasm with which we were received everywhere was exhilarating. In every town, in every village, at each detached farmhouse, the people assembled to cheer us, to shake us by the hand, and to give gifts. They gave us coffee, bread, curds, cheese, fruit, wine, envelopes, handkerchiefs—all sorts of things like that. And how they hate and loathe the Germans! We billeted that night in a small town not far from the Belgian frontier, with hopes of a long night to make up for our previous hard days. But we were destined to disappointment. Next morning at 3.30 a.m. I was afoot, trying to find the whereabouts of my men in a strange place. By a mistake they had been placed in other billets than those indicated at first. I found them at last sleeping like logs in the hay in two large barns. That day, at the end of the march, the A.D. of M.S. came to us and said that no other field ambulance than ours had yet got up, so we were to be split into three sections, and each section to be attached to a brigade. My section I took off in a direction indicated, and reported myself to my brigade headquarters. I was billeted comfortably in a house, the people of which vied one with the other in efforts to make us comfortable. The lady of the house herself assisted at the cooking of a meal for us, but I fancy the middle-class French woman usually does assist the cook. We had an excellent meal, one of the guests being a French officer.

### The Battle of Mons.

I got orders to march at 4.30 a.m., and did so. That day we crossed into Belgium, and came to a halt near Mons in a considerable industrial district. We were told we might expect battle next day. Already we had been meeting the stream of refugees—the saddest sight of war, a constant stream of poor people steadily flowing west, drifting along carrying small possessions. Many women with children in perambulators, some with babies at the breast; old men, cripples, richer persons in carts and wagons, but all flying sadly and wearily before the Germans or drawing to shelter and succour in the west because their homes already were destroyed. The people were most friendly, only too willing to do all that lay in their power for "les Anglais." I was told by authority to take such houses as I required for my purposes in a certain street on the outskirts of the town. I took over parts of enough houses to accommodate my men on either side of other houses of which I prepared the ground floors for hospital purposes. I put in these such sick as I had collected on the way.

### A Convent Red Cross Hospital.

The vehicles of my section I parked in column, head to the road, ready to draw out at any minute. The horses I picketed each pair by its wagon, with the harness beneath the wagon. I placed a guard, saw all was in order, and turned in to sleep on a stretcher when I was satisfied all was right. I was up early next morning, and placed all my sick in a Red Cross detachment hospital formed in a convent, because I wished to be quite clear for wounded. I believe the lot were killed in the hospital that night. I packed my wagons up completely ready to move, but made the inhabitants get ready hot water, basins, towels, and all things I could think of ready to my hand. I wished to be able to move to another part of the battlefield at short notice, and yet to be so ready that I could open a hospital where I was in twenty minutes.

### The German Attack.

Meanwhile at the first sign of dawn the German attack had begun. I could see the puffs of smoke arising where our guns were in action—north-west, north, and north-east of us—and could hear the sounds of our cannonade, see and hear the burst of German shell, and hear a furious battle of musketry. So I flew the Union Jack and the Red Cross, and sat down to wait events. So we sat and had breakfast and had dinner, with no other excitement than the noise of the firing and the passing over of aeroplanes. At intervals I sent to brigade headquarters, about 700 yards away, to get information and to ask if I was wanted anywhere. I gathered that the British were making a most gallant stand against a vastly superior force, and that the list of casualties was heavy, and that the issue was more than doubtful. I heard tales of incidents of the fight, such as that one of our heavy batteries had smashed a German battery in eight minutes till you could find scarcely the scraps, and that we had had heavy loss in a certain part of the field north-east from me.

### Preparing for the Wounded.

Quite suddenly in the middle of the afternoon I got orders to move to a certain point indicated to me on a map. I pulled down my flag and moved off. When I was sure I was on a line that prevented the possibility of my section straying, I rode forward to reconnoitre my ground. It was not my choice, nor did I like the place, because the presence of railways on three sides interfered with my freedom of movement. There was a small field, reached by a roadway through a small allotment garden. This was the only place suitable to park my wagons, so I ordered the section to follow me in as soon as it came up, and by marching it round the field placed it in column under shelter of a bank and so placed that I could draw out rapidly without confusion. I ordered my men to remain under the bank, except such as I told off to water the horses and set up my flags, and, satisfied that my dispositions were the best possible, I sat down myself on a place from which I could keep all under my eye. The people from the town now came streaming in in hundreds, bringing coffee and curds and bread and other foods. We sat and fed sumptuously while the cannon roared and musketry rattled very near at hand, but we could see nothing except a quantity of our artillery moving along a ridge about half a mile away and a large number of men, Belgian civilians, digging trenches and burning crops between us and them. I sent a party of men to the crops with orders to bring in as many sheaves of oats as possible and to set them before the horses. I may say here I believe it was only the knowledge I have of horses and the care I took to see that they were stuffed with food and water on every possible occasion that enabled them to do the enormous quantity of work I got out of them during the next few days.

### Terrific Fire and Fears of Panic.

We were all settled quietly here when of a sudden on the other side of the road I saw shells bursting in the air, numbers of them, little clouds of white smoke suddenly appearing one after the other quickly and lingering, so that many little clouds were in the air before the first had dissolved. Whilst we watched shells began to burst over the Belgians in the trenches. I watched expecting to see many men fall. Of course they fled, and so did our civilian friends who were feeding us, and the shells burst over them all the while, but, and it is a most curious thing, not a single man fell. Many came back through my field. All assured us no one was hurt. In a few minutes we were alone. I feared lest my men might get panic, so I fell them in, told them to lie down, and stood by them myself smoking and making jokes about the hurry of the civilians, but I did not like my position. I could hear the battle was raging furiously across the road hidden from me by some large works of sorts. Of a sudden the din increased. The musketry fire was terrific; I could hear shoutings and the tramp of feet and the sound of men running. I felt sure a great deal was happening very close to me. I felt uneasy. I knew our men were being forced back, so I fell in my men and told them that I was going to move the section till the head of my column was on the road so that I might be able to get out of the field without confusion if I had to do so. I went ahead, of course, and placed one officer in the rear.

### A Surprise and a Risky Advance.

The other I had sent to ascertain the position of a certain hospital in the town. I had not been standing on the road many minutes before I saw soldiers running up it and a staff officer I knew riding up. I stopped the latter and said, "What is up?" He said, "I do not know; our fellows broke and ran like rabbits." I may say that a certain regiment, after maintaining a most gallant fight all day, got outflanked and were making an orderly retirement, when they took a road that was too much to the east and marched in close order right into a strong German force that was lying hidden. They were surprised and shot down in large numbers. They broke, and now were flying back up the road on which I stood pursued by the Germans. I could see a crowd of men coming. Before them came a general looking worried. He said to me, "You are badly wanted down there." I did not think "down there" looked a very good place to be, but I at once ordered my bearer sub-division to advance down the road 100 yards and halt, and my tent sub-division to advance across the road and halt. I then sent two ambulance wagons after the bearer sub-division, and sent the other ambulance wagons and all the carts 100 yards in the other direction. You see I thus had my bearers ready to get forward, my tent division ready to form a dressing station on an open space by the road, and all my other wagons headed on the line of retreat. This only took a few seconds and a few words to arrange. I went down to the bearer sub-division and ordered the officer in charge to advance cautiously down the street and to collect such wounded as he could. The stream of fugitives was pouring by us, some wounded but able to walk. I impressed on my officer the importance of as much caution as possible, saw him start, and came back to my dressing station.

### The Nerves of Men and Wounded.

Already in these few minutes some slightly wounded men had come there and stopped, and other men not wounded, but highly excited or exhausted, had thrown themselves down when they saw a place for medical attention. Many of them were being violently sick from no other cause than excitement and funk. I set to to dress the wounded and to send on the others up the street after their comrades. Some of the wounded took things quietly, some were in a terrible funk. One man, who had no other injury than a graze, where a bullet had gone between his fingers, threw himself down on the steps of a small house. His hand was covered with blood. A corporal went to dress him. I followed after. We could not ascertain the extent of his injury at first, because every time the sound of fire got louder, or a bullet came up the street, he would jump up and yell, "Oh! they are coming, they are coming." The orderly and I talked to him, now soothing him as if he were a baby, and now cursing him for a fool. We collected all the wounded and placed them in the reserve ambulance wagon, except the very slightly hurt. Of course, all these were men able to walk; the more severe cases were lying on the ground beyond us where they had fallen. The fugitives had disappeared. An incautious Belgian woman came out of a house and got a German bullet in her ankle. The quartermaster-sergeant said to me, "There are only three soldiers in sight in the street now." The street was empty excepting for my parties and the wounded I had collected.

### A Brave Corporal.

I got all the rest of the wounded into my wagons, and ordered the quartermaster-sergeant to take charge of the wagons and the men of the tent sub-division, and to mount them up to the top of the hill and halt just over the brow. I said I'd stay back alone to get back the other party, about whom I was feeling anxious. At this time there was hot firing going on all round me, but yet I could not see a man—the banks, hedges, and railways, etc., hid all from me. It struck me that if I got shot there might be no one to do anything or to know what to do, so I called to Corporal Chatting, whom I had observed to be braver than most. My men were all doing well; none showed the "white feather." There was no confusion. But this man I had noticed particularly, as he helped me with some frightened men in the street. He came back to me. I told him I should keep him with me. He was pleased, and evidently appreciated it as a compliment. We stood alone in the street. I rolled a cigarette and had a smoke, while I rapidly gave him directions in case of trouble. We then strolled quietly down the road till I saw the tail of the rear ambulance wagon. I stood a minute watching, then I said to Chatting, "I'll go on and tell them to come back."

### Shelling the Hospital.

To my joy I saw the head of the horses of the other wagon come round the corner behind the rear one. I hailed them as they came. To avoid panic I collected the men together, got them into fours, put myself at their head, and made them march in step up the deserted place. I called on those who could whistle to whistle a march, and we swung up the street in perfect order. Wonderful to relate, not a man or horse was touched. We had our wagons full of wounded. The Germans were held in check apparently now, as the firing got less intense. I expect they feared to rush the town at once lest they should find they were in a trap. I took all the wounded to a civil hospital in the town run by Red Cross people and hurried them into it. I fear the Germans killed them all after we had gone, as I hear they shelled the place and burnt it. I then got all the wagons on the line of retreat, but heard from an officer that there were wounded down a certain way in the direction of some firing. It did not seem much good to take my command on into danger for wounded I was not certain about, so I took Chatting, and he and I explored the deserted streets, but saw no one sick or sound, so we started off to collect wounded as we went and to follow the retreat.

